# Socio-demographics and asthma prevalence, management, and outcomes among children 1–11 years of age in California

**DOI:** 10.1186/s41256-021-00199-y

**Published:** 2021-05-27

**Authors:** Jim E. Banta, Majed Ramadan, Noara Alhusseini, Khaled Aloraini, Naomi Modeste

**Affiliations:** 1 Loma Linda University School of Public Health, 24951 Circle Dr, CA 92354 Loma Linda, USA; 2 College of Medicine, Alfaisal University, 7746 Ibrahim Alziady St., Alwurud District, 12253 2499 Riyadh, Saudi Arabia

**Keywords:** Children, Ethnic groups, Parents, Asthma, Cross-sectional studies, Surveys and Questionnaires, Language, California

## Abstract

**Background:**

Asthma disproportionately affects minority and low-income children. We examined asthma prevalence, management and outcomes, focusing on race/ethnicity and acculturation of parents (particularly English language proficiency).

**Methods:**

This cross-sectional, correlational analysis used a de-identified population-based survey, the California Health Interview Survey, for years 2001–2015. Survey-weighted analysis with SAS 9.4 was used to determine asthma prevalence among children 1 to 11 years of age. Descriptive analysis was conducted, adjusting for survey design and combination of multiple years of data. The Pearson test, using design-based F values was used to determine statistically significant differences between those having/not having a doctor diagnosis of asthma. Multivariable logistic regression, with jackknife approach to obtain confidence intervals, was used to examine associations of child and parental characteristics with asthma prevalence, management, and outcomes.

**Results:**

The 61,625 completed surveys represented an estimated annual population of 5.7 million children, of which 12.9 % had asthma. There were significant (*p* < 0.001) differences by age, gender, race, and language proficiency, with higher asthma prevalence for children 6 to 11 years of age (15.5 %), males (15.3 %), African Americans (19.5 %), and parents speaking English very well (14.1 %). Compared to children whose parents spoke English very well, those whose parents spoke English not well or not at all were less likely to achieve optimal asthma management, i.e. to have received a management plan from doctor (OR 0.30; 95 % Confidence Interval 0.20–0.46)), to be currently taking medication to control asthma (OR 0.52; 95 % CI 0.36–0.74)), or to be not confident in ability to control asthma (OR 3.10; 95 % CI 1.49–6.42). Children whose parents spoke English fairly well rather than very well had worse outcomes, i.e. were more likely to have an emergency room visit in past 12 months (OR 1.92; 95 % CI 1.03–3.61) and were more likely to miss school due to asthma in past 12 months (OR 0.71; 1.01–2.94).

**Conclusions:**

Socio-demographics had a limited role in explaining differences across a handful of asthma management and outcome measures in California. Parental English language proficiency had the most consistent influence, underscoring the need for culturally and linguistically competent care.

## Background

Asthma is a leading chronic disease among children in the United States [1], with prevalence being higher among boys (9.2 %) than among girls (7.4 %) [2]. Prevalence is higher among minorities [3], particularly among non-Hispanic African American/Black children (15.7 %) compared to white children (7.1 %) [2]. Uncontrolled asthma symptoms can lead to serious complications ranging from sleep disturbance [4] to death [5]; thus, the imperative to address determinants of asthma burden among children [6]. A recent CDC study found that 51 % of children in school with current asthma missed at least one school day as a result of asthma in the prior 12 months [7]. Asthma, particularly poor asthma control, is associated with more emergency department (ED) visits [8] and increased hospitalization. Health care expenditures in the United States are estimated to be $5.92 billion for treatment among school-aged children with asthma, which represents an extra $847 expenditures per child as a result of asthma [9].

Asthma in children is the result of complex gene-environment interactions, with some risk factors including sex (boys), parental history of asthma, and personal history of allergy [10, 11]. Additional risk factors for asthma include air pollution, psychosocial stress, obesity and inadequate treatment [12, 13]. Barriers to receiving adequate treatment include economic disadvantages, lack of education, limited or lack of health insurance plans and poor access to medication [13]. Asthma is more common among children and families below poverty level [14]. There is a significant association between air pollution and impaired lung functions among minorities with asthma [15]. Furthermore, childhood asthma is more common within public housing compared to private housing due to multiple housing quality factors [16].

Children with asthma report cost barriers to healthcare services such as the inability to afford medication and see a primary physician. A California study found that 30 % of asthma control prescriptions prescribed to low-income children were never filled [17]. A related barrier is lack of health care insurance [13] or public insurance, particularly among Latino children [18].In a study of children having the same access to healthcare, Black and Hispanic children were more likely to have avoidable hospitalizations, asthma-related ED visits, and less likely to visit a specialist compared to white children [19]. Black, Hispanic, and low-income children with asthma are more likely to not have a usual source for medical care and more likely to use EDs than are white children [20].

Adverse asthma outcomes can be managed by controlling environmental measures, increased patient education and medication [17]. Furthermore, care coordination, self-management education and patient-centered approaches play a vital role in controlling asthma and limiting healthcare utilization [21]. Barriers that limit participation in asthma management programs include poverty, language barriers, lack of insurance, and difficulty in arranging transportation to physicians’ clinics [22]. Using alternative medicine and home remedies among minority groups can alter the effectiveness of medication, interact with treatment outcomes and cause a harmful effect [23].

The goal of this study was to examine asthma prevalence, management, and outcomes among California children. Of particular interest is examining the influence of race/ethnicity and acculturation-related measures with respect to asthma prevalence, management, and outcomes.

## Methods

### Data Source

The California Health Interview Survey (CHIS) is considered to be the largest state health survey in the nation [24]. It began as a biennial population-based survey in 2001 and is collaboratively facilitated by the University of California, Los Angeles Center for Health Policy Research and other state agencies. It is conducted in languages such as English, Spanish, Cantonese, Mandarin, Korean, and Vietnamese [25]. Public-use files are released for children (0–11 years of age), adolescents (12–17 years of age), and adults (18 years of age and greater).

There were nine different public-use data survey files, which were downloaded directly from the UCLA website and combined for this analysis. CHIS initially released data in two-year increments, switching to one-year data files in 2013. Different weighting variables were used for each survey year, and survey year was included as a covariate in all multivariable regressions. Though many questions were asked consistently throughout the study period, there were also changes over time. Thus not all years of data were used for some analysis of outcomes. Sometimes there were differences in how data was reported. For example, parental education had slightly different levels of detail depending on survey year. However, after recoding to fewer groups, there was equivalence over time.

### Sample

CHIS is a population-based telephone survey that uses a random-digit-dial sample for both landlines and cell phones, with the landline sample segregated into 56 geographic sampling strata, corresponding to county boundaries within the state. From each stratum, residential telephone numbers were selected to interview an adult. If the household included a child or adolescent, the adult was also asked questions about that child and/or adolescent. If there were multiple children or adolescents, CHIS randomly selected one for survey purposes. The sampling design began including cell phones in 2009, with percentages steadily increasing over time. For example in CHIS 2013–2014, 19.3 % of adult surveys were conducted using cell phones, compared to 46.2 % in CHIS 2015[26]. Survey completion rates from CHIS are comparable to other large-scale surveys, such as the California Behavioral Risk Factor Surveillance System [27]. In 2009, the screening completion rate of all households was 35.1 % and the child extended survey completion rate was 72.9, for a survey completion rate of 26.3 % for child surveys [27]. As a result of the sampling design, users are able to make population estimates. Researchers have demonstrated that CHIS population estimates are consistent with California’s official demographic estimates when stratified by a number of demographic characteristics, including gender, race/ethnicity, and age group [28]. For this study we examined children data for 2001–2015, dropping infants (0 years of age) from analysis since their parents were not asked about many health topics.

### Measures

Asthma prevalence was determined by the question “Have you ever been told by a physician that you have asthma?” Subsequent analyses were restricted to those reporting ‘yes’. Asthma management measures included taking daily medication to control asthma (yes/no), doctor ever give management plan for child (yes/no), have a written copy of a plan (yes/no), and confidence to control asthma (yes/no). Asthma outcome measures were: asthma attacks last 12 months [numeric, recoded (yes/no)], emergency department visits last 12 months – for any reason [numeric, recoded (yes/no)], and missed school due to asthma in last 12 months (yes/no). Not all asthma-related questions were asked during every survey year.

There were two measures of acculturation. One was citizenship status of father (US born, naturalized, and undocumented immigrant). The other was language, as it has been identified in the literature as a rational proxy for acculturation for Latinos [29]. Adult level of English proficiency was self-reported as very well, fairly well and not well / not at all (the last two combined by authors). English language proficiency has been used in national surveys to study access to care [30].

Demographics included age of child (categorized for this study as 0–5, and 6–11 years), gender of child, and race/ethnicity of child (white, Latino, Black, Asian, and “other”, which included “Native Hawaiian” and “Two or more races”). Socioeconomic status was assessed based on education level and poverty level using CHIS variables of (a) adult education attainment categorized as an ordinal scale and (b) poverty level (ordinal scale). Geographic location was based on respondent zip code and categorized by UCLA into urban or rural. Healthcare access measures included: (a) usual source of care (ordinal) and (b) any insurance in the last 12 months (nominal as provided by CHIS).

### Data Analysis

All data analyses were conducted using SAS 9.4 software (SAS Institute, Cary, NC). Descriptive analysis was conducted to obtain frequencies for all variables, adjusting for survey design and combination of multiple years of data. The Pearson test, using design-based F values, for categorical variables was used to determine statistical significant differences between each category. To analyze the association between race and acculturation with asthma prevalence and outcomes, multivariable logistic regression was conducted, and a jackknife approach used to obtain confidence intervals. The jackknife approach to variance estimation is preferred for CHIS data since they provide 80 replicated weights per survey year in order to ensure that the sampled data best matches the California population [31]. Asthma management and outcome analyses were conducted only for children diagnosed with asthma. All analyses used survey-specific routines to account for the complex sampling design for each survey year. This research was considered exempt from university IRB approval due to the de-identified nature of the public use data.

## Results

As seen in Table 1, there were 61,625 completed surveys, representing an estimated annual population of 5.7 million children between 2001 and 2015. Of these, 12.9 % reported having asthma at the time of survey. There were significant differences (*p* < 0.001) for child’s age, gender, race, and parent’s education, citizenship status, and English language proficiency. Asthma prevalence was higher among children 6 to 11 years of age (15.5 %), males (15.3 %), African Americans (19.5 %) and those of two or more races (13.7 %), children whose parents were a high school graduate (13.4 %), had some college (16.1 %), who spoke English very well (14.1 %), or was a US-born citizen (14.2 %). Prevalence was lowest among whites (12 %) and those with no usual source of care (7.4 %). There were also significant differences based on usual source of care (*p* = 0.0045), with higher prevalence among those normally receiving care at a doctor’s office/HMO/Kaiser (13.3 %) and insurance (*p* = 0.022), with higher prevalence among the insured (13.0 %). There were not significant differences based on poverty level, rural/urban status, or survey year.

**Table 1:** Survey-weighted characteristics of Hispanic and White California children, 2001 to 2015

	**Total n (percent)**	**Percent with Asthma, CI **	Asthma *P*-value	
	61,625 (100%)	12.9 (12.3, 13.4)		
Age			< 0.001	***
1 to 5 years	26,856 (45.2%)	9.7 (8.9, 10.4)		
6 to 11years	34,769 (54.8%)	15.5 (14.7, 16.4)		
Gender			< 0.001	***
Male	31,684 (51.0%)	15.3 (14.5, 16.1)		
Female	29,941 (49.0%)	10.4 (9.5, 11.1)		
Race/ethnicity			< 0.001	***
Hispanic	18,764 (42.1%)	12.4 (11.3, 13.5)		
Asian	6,627 (10.7%)	12.9 (10.8, 14.9)		
African American	2,801 (6.6%)	19.5 (16.8, 22.2)		
White	29,979 (34.5%)	12.0 (11.3, 12.8)		
Two or more Races	4,454 (6.2%)	13.7 (11.3, 16.1)		
Adult English Proficiency			< 0.001	***
Very well	43,953 (66/2%)	14.1 (13.2, 14.9)		
Fairly well	6,349 (12.4%)	11.9 (9.9, 14.0)		
Not well / Not at all	11,323 (21.4%)	9.7 (8.6, 10.8)		
Adult Education			< 0.001	***
Less than 12 years education	10,443 (21.4%)	11.0 (9.9, 12.1)		
High School graduate	13,247 (22.4%)	13.4 (11.9, 14.9)		
Less than 4 years college	14,369 (22.2%)	16.1 (14.7, 17.5)		
College graduate or higher	23,568 (34.1%)	11.4 (10.5, 12.3)		
Poverty level			0.6217	
0-99% FPL	10,389 (24.0%)	13.3 (11.8, 14.8)		
100-199% FPL	12,460 (22.5%)	13.2 (11.9, 14.4)		
200-299% FPL	8,635 (13.7%)	13.1 (11.5, 14.7)		
300% FPL and Above	30,141 (39.8%)	12.4 (11.6, 13.2)		
Living environment			0.0680	
Urban	50,563 (89.2%)	13.0 (12.4, 13.7)		
Rural	11,064 (10.8%)	11.5 (10.1, 12.9)		
Usual Source of Care			0.0045	**
Doctor office/HMO/Kaiser	45,244 (69.2%)	13.3 (12.6, 14.0)		
Community/Government clinic	14,208 (22.7%)	12.5 (11.2, 13.7)		
ER/Urgent care/ Other place	550 (1.0%)	12.6 (8.1, 17.1)		
No usual source of care	1,623 (3.1%)	7.4 (5.0, 9.8)		
Insurance status			0.0222	*
Insured	58,430 (95.1%)	13.0 (12.4, 13.6)		
Not insured	3,195 (4.9%)	9.8 (7.3, 12.2)		
Citizenship Status			< 0.001	***
US-born citizen	38,019 (55.7%)	14.2 (13.3, 15.0)		
Naturalized citizen	10,014 (17.4%)	12.2 (10.8, 13.7)		
Non-Citizen	13,592 (26.7%)	10.6 (9.5, 11.7)		
Survey year			0.2457	
2001-2002	11,794 (11.2%)	12.7 (12.0, 13.5)		
2003-2004	7,863 (11.2%)	13.7 (12.7, 14.8)		
2005-2006	10,403 (11.4%)	13.5 (12.4, 14.6)		
2007-2008	9,134 (11.5%)	13.5 (12.4, 14.5)		
2009-2010	8,343 (11.5%)	11.2 (10.8, 12.2)		
2011-2012	6,879 (10.9%)	14.2 (12.7, 15.7)		
2013	2,763 (10.7%)	13.6 (11.2, 15.9)		
2014	2,457 (10.8%)	12.0 (9.4, 14.6)		
2015	1,989 (10.9%)	11.5 (8.4, 14.6)		

Estimated annual population of children = 5,697,515. Those with asthma = 733,320. Sample numbers are unweighted, all percents are survey-weighted

*CI* 95% Confidence Interval

* *p*<0.05, ** *p*<0.01, *** *p*<0.001

As seen in Table 2, many of these findings identified in bivariate comparisons were confirmed in regression analysis. Particularly significant findings for age, gender, race, and English language proficiency and non-significant findings for survey year. Changes in regression included insurance and citizen status becoming statistically insignificant. However, there were now significantly lower likelihoods of having an asthma diagnosis for those at or above 200 % of federal poverty level and for those living in a rural environment (odds ratio 0.85; 95 % CI 0.74–0.99). A nuance for parental education was that children whose parents had a high school degree were not significantly more likely to have asthma, only those whose parents had some college.

**Table 2:** Logistic Regression for asthma diagnosis (*n* = 61,625)

	Doctor Ever Told you Your Child has Asthma		
	OR	95 % CI	*P*-value
Age			
1 to 5 years (ref.)	---	---	
6 to 11years	1.78***	(1.60, 1.99)	< 0.001
Gender			
Male (ref.)	---	---	
Female	0.63***	(0.57, 0.70)	< 0.001
Race/ethnicity			
Hispanic (ref.)	---	---	
Asian	1.06	(0.85, 1.32)	0.595
African American	1.17*	(1.02, 1.50)	0.299
White	0.68***	(0.61, 0.75)	< 0.001
Two or more Races	0.93	(0.59, 0.79)	0.634
Adult English Proficiency			
Very well (ref.)	---	---	
Fairly well	0.78*	(0.61, 1.02)	0.047
Not well / Not at all	0.60***	(0.48, 0.77)	< 0.001
Adult Education			
Less than 12 years (ref)	---	---	
High School graduate	1.04	(0.86, 1.26)	0.682
Some college	1.21*	(1.00, 1.46)	0.045
College graduate or higher	0.91	(0.74, 1.11)	0.356
Poverty level			
0–99 % FPL (ref.)	---	---	
100–199 % FPL	0.85	(0.71, 1.04)	0.159
200–299 % FPL	0.77*	(0.61, 0.96)	0.021
300 % FPL and Above	0.74**	(0.55, 0.83)	0.005
Living environment			
Urban (ref.)	---	---	
Rural	0.85	(0.74,0.99)	0.038
Usual Source of Care			
Office/HMO/Kaiser (ref.)	---	---	
Government/community	1.00	(0.86, 1.16)	0.964
ER/Urgent care/ Other place	0.88	(0.55, 1.42)	0.582
No usual source of care	0.57**	(0.40, 0.84)	0.004
Insurance status			
Insured (ref.)	---	---	
Not insured	0.79	(0.60, 1.07)	0.125
Citizenship Status			
US-born citizen (ref.)	---	---	
Naturalized citizen	0.85	(0.71, 1.03)	0.146
Non-Citizen	0.82	(0.66, 1.01)	0.066
Survey year			
2001–2002 (ref)	---	---	
2003–2004	1.11	(0.99, 1.25)	0.075
2005–2006	1.09	(0.97, 1.23)	0.128
2007–2008	1.08	(0.96, 1.22)	0.187
2009–2010	0.89	(0.78, 1.02)	0.109
2011–2012	1.12	(0.97, 1.31)	0.108
2013	1.05	(0.85, 1.30)	0.664
2014	0.89	(0.69, 1.16)	0.393
2015	0.85	(0.62, 1.18)	0.338

*OR *Odds Ratio, *CI *Confidence Interval

* *p* < 0.05, ** *p* < 0.01, *** *p* < 0.001

Table 3 presents results for four measures of asthma management. The most notable finding is that parental language proficience was significantly associated with three of the outcomes. Compared to those with English language proficiency of very well, both those with proficience of fairly well and not well/not at all were less likely to be taking daily medications to control asthma and to have been given an asthma management plan by a doctor, and the parents were more likely to not be confident that they could not control asthma.

**Table 3:** Logistic Regression for Asthma Management

	Currently Taking Daily Medication to Control Asthma	Doctor Ever Given Asthma Management Plan for Child	Have Written Copy of Plan	Not Confident can Control Asthma
	*n*=6,669	*n*=6,117	*n*=2,026	*n*=2,836
Proportion	32.6% (30.8, 34.4)	60.3% (57.5, 63.2)	32.3% (28.1, 36.5)	27.2% (23.1, 31.4)
Age				
1 to 5 years (ref.)	---	---	---	---
6 to 11years	0.74 (0.62, 0.89)**	0.91 (0.73, 1.14)	0.71 (0.47, 1.08)	0.72 (0.48, 1.10)
Gender				
Male (ref.)	---	---	---	---
Female	0.85 (0.72, 1.02)	1.04 (0.80, 1.34)	1.28 (0.85, 1.93)	1.06 (0.68, 1.63)
Race/ethnicity				
Hispanic (ref.)	---	---	---	---
Asian	0.57 (0.39, 0.82)**	0.76 (0.47, 1.22)	0.86 (0.44, 1.69)	0.53 (0.21, 1.32)
African American	0.98 (0.71, 1.36)	0.84 (0.54, 1.32)	1.10 (0.52, 2.33)	1.45 (0.69, 3.02)
White	0.65 (0.51, 0.83)**	0.88 (0.63, 1.23)	1.26 (0.73, 2.17)	0.91 (0.54, 1.56)
Two or more Races	0.90 (0.59, 1.38)	0.62 (0.38, 1.03)	1.11 (0.49, 2.51)	0.70 (0.35, 1.42)
Adult English Proficiency				
Very well (ref.)	---	---	---	---
Fairly well	0.63 (0.45, 0.87)**	0.45 (0.25, 0.80)**	0.54 (0.23, 1.24)	3.39 (1.52, 7.54)**
Not well / Not at all	0.52 (0.36, 0.74)***	0.30 (0.20, 0.46)***	1.34 (0.58, 3.12)	3.10 (1.49, 6.42)**
Adult Education				
Less than 12 years (ref)	---	---	---	---
High School graduate	1.13 (0.85, 1.52)	1.25 (0.82, 1.92)	1.55 (0.75, 3.21)	1.07 (0.52, 2.22)
Some college	0.94 (0.69, 1.27)	1.33 (0.84, 2.11)	2.33 (1.07, 5.08)*	1.03 (0.45, 2.34)
College graduate or higher	0.93 (0.68, 1.27)	1.94 (1.25, 3.02)*	3.21 (1.35, 7.64)**	0.95 (0.43, 2.08)
Poverty level				
0-99% FPL (ref.)	---	---	---	---
100-199% FPL	0.73 (0.56, 0.96)*	1.04 (0.70, 1.53)	0.98 (0.54, 1.78)	0.58 (0.32, 1.08)
200-299% FPL	0.72 (0.51, 1.03)	0.99 (0.55, 1.77)	1.02 (0.51, 2.01)	1.43 (0.58, 3.50)
300% FPL and Above	0.63 (0.47, 0.85)**	1.03 (0.67, 1.58)	0.62 (0.33, 1.17)	0.89 (0.44, 1.81)
Residence				
Urban (ref.)	---	---	---	---
Rural	1.12 (0.87, 1.45)	1.15 (0.83, 1.58)	1.03 (0.58, 1.84)	1.23 (0.63, 2.41)
Usual Source of Care				
Office/HMO/Kaiser (ref.)	---	---	---	---
Government clinic / Community hospital	0.75 (0.61, 0.92)**	0.99 (0.72, 1.38)	1.64 (0.93, 2.91)	0.98 (0.60, 1.58)
ER/Urgent care/ Other place	0.54 (0.26, 1.12)	1.26 (0.63, 2.55)	0.11 (0.02, 0.50)**	0.54 (0.10, 2.84)
No usual source of care	0.48 (0.24, 0.98)*	0.30 (0.12, 0.74)*	1.75 (0.25, 12.08)	1.54 (0.36, 6.60)
Insurance status				
Insured (ref.)	---	---	---	---
Not insured	0.86 (0.56, 1.31)	0.88 (0.53, 1.49)	2.25 (0.36, 14.11)	0.61 (0.20, 1.86)
Citizenship Status				
US-born citizen (ref.)ta	---	---	---	---
Naturalized citizen	1.10 (0.83, 1.46)	1.10 (0.73, 1.67)	1.73 (0.92, 3.26)	1.20 (0.65, 2.21)
Non-Citizen	1.23 (0.91, 1.67)	1.48 (0.90, 2.44)	1.27 (0.58, 2.36)	1.45 (0.69, 3.05)
Survey year				
2001-2002	REF	N/A	N/A	NA
2003-2004	0.58 (0.46, 0.75)***	REF	N/A	N/A
2005-2006	0.37 (0.29, 0.48)***	1.01 (0.78, 1.30)	N/A	N/A
2007-2008	0.39 (0.30, 0.51)***	0.98 (0.77, 1.24)	N/A	N/A
2009-2010	0.50 (0.38, 0.66)***	2.74 (2.13, 3.52)***	REF	REF
2011-2012	0.38 (0.28, 0.53)***	3.42 (2.47, 4.73)***	1.32 (0.90, 1.96)	0.81 (0.53, 1.23)
2013	N/A	2.78 (1.57, 4.92)***	0.99 (0.61, 1.62)	0.87 (0.51, 1.49)
2014	N/A	N/A	1.84 (1.13, 2.98)*	0.93 (0.53, 1.64)
2015	N/A	2.16 (1.08, 4.34)*	1.35 (0.64, 2.82)	0.74 (0.34, 1.65)

* *p* < 0.05, ** *p* < 0.01, *** *p* < 0.001

N/A-question not asked during that survey year

With regards to other covariates, gender, rural/urban, insurance, and citizenship status were not significantly associated with any asthma management measure. Race (Asian and white less likely), poverty (less likely if not below 100 %FPL), source of care (those with no usual source and those seen in government clinic/community hospital less likely), and survey year (decrease over time) were significantly associated with daily use of asthma medication. Age (older children less likely), parent’s eduation (college graduates more likely), source of care (no usual source less likely), and survey year (increase over time) were significantly associated with doctor ever gave an asthma management for child. Parent’s education (college more likely) and source of care (ER/urgent care less likely) were significantly associated with parent currently having a written copy of asthma management plan. No other measure besides adult language proficiency was significantly associated with confidence to control asthma.

Table 4 presents results of three asthma outcome measures. English language proficiency was significantly associated with two of the measures: children whose parent’s were able to speak fairly were more likely to have an emergency room visit in past 12 months and to have missed school in past 12 months due to asthma. Many variables were not statistically significant with any outcome: gender, poverty, living environment, insurance, and citizenship status. Children 6–11 years of age were less likely to have any of all three outcomes. African Americans were more likely to have an emergeny room visit. Children whose parents had some college were more likely to have an asthma attack in the past 12 months. Emergency room visits were generally lower over time.

**Table 4:** Logistic Regression for Asthma Outcomes

	Had Asthma Attack In Past 12 Months	Emergency Room visit in past 12 months	Missed School due to Asthma in Past 12 Months
	*n*=5,514	*n*=5,339	*n*=6,435
Proportion	45.2% (43.0, 47.4)	30.9% (28.0, 33.8)	32.8% (30.4, 35.2)
Age			
1 to 5 years (ref.)	---	---	---
6 to 11years	0.62 (0.51,0.74)***	0.44 (0.33, 0.57)***	0.79 (0.63, 0.99)*
Gender			
Male (ref.)	---	---	---
Female	0.90 (0.76, 1.07)	1.02 (0.74, 1.43)	1.08 (0.84, 1.39)
Race/ethnicity			
Hispanic (ref.)	---	---	---
Asian	0.94 (0.63, 1.40)	0.62 (0.36, 1.07)	0.71 (0.45, 1.12)
African American	0.95 (0.65, 1.40)	2.10 (1.24, 3.55)**	1.55 (0.95, 2.52)
White	1.14 (0.88, 1.49)	0.99 (0.70, 1.41)	0.75 (0.54, 1.04)
Two or more Races	0.94 (0.62, 1.42)	0.97 (0.55, 1.72)	0.65 (0.40, 1.06)
Adult English Proficiency			
Very well (ref.)	---	---	---
Fairly well	0.86 (0.60, 1.24)	1.92 (1.03, 3.61)*	1.73 (1.01, 2.94)*
Not well / Not at all	0.74 (0.51, 1.07)	0.89 (0.51, 1.55)	1.08 (0.69, 1.70)
Adult Education			
Less than 12 years (ref.)	---	---	---
High school graduate	1.16 (0.83, 1.64)	1.30 (0.73, 2.28)	0.90 (0.61, 1.34)
Some college	1.42 (1.01, 1.98)*	1.52 (0.91, 2.53)	0.83 (0.56, 1.23)
College graduate or higher	1.33 (0.93, 1.90)	1.06 (0.62, 1.78)	0.71 (0.46, 1.08)
Poverty level			
0-99% FPL (ref.)	---	---	---
100-199% FPL	1.04 (0.80, 1.34)	0.84 (0.48, 1.46)	1.07 (0.71, 1.60)
200-299% FPL	1.18 (0.83, 1.68)	0.79 (0.45, 1.38)	1.32 (0.79, 2.21)
300% FPL and Above	1.18 (0.88, 1.59)	0.86 (0.55, 1.33)	1.07 (0.71, 1.64)
Living environment			
Urban (ref.)	---	---	---
Rural	1.24 (0.97, 1.58)	1.38 (0.92, 2.07)	1.04 (0.75, 1.44)
Usual Source of Care			
Office/HMO/Kaiser (ref.)	---	---	---
Government clinic / Community hospital	0.86 (0.71, 1.06)	0.98 (0.67, 1.44)	0.91 (0.68, 1.21)
ER/Urgent care/ Other	0.39 (0.19, 0.78)**	2.80 (1.27, 6.17)*	0.69 (0.30, 1.57)
No usual source of care	0.60 (0.32, 1.12)	1.16 (0.41, 3.22)	0.66 (0.27, 1.62)
Insurance status			
Insured (ref.)	---	---	---
Not insured	0.83 (0.51, 1.36)	0.64 (0.38, 1.06)	0.88 (0.41, 1.88)
Citizenship Status			
US-born citizen (ref.)	---	---	---
Naturalized citizen	1.16 (0.84, 1.60)	1.08 (0.70, 1.73)	0.88 (0.60, 1.31)
Non-Citizen	0.91 (0.65, 1.26)	1.22 (0.73, 2.05)	0.82 (0.53, 1.27)
Survey year			
2001-2002	N/A	N/A	N/A
2003-2004	REF	N/A	REF
2005-2006	1.06 (0.86, 1.32)	REF	1.14 (0.91, 1.43)
2007-2008	0.91 (0.71, 1.16)	0.75 (0.58, 0.98)*	1.05 (0.81, 1.35)
2009-2010	0.93 (0.72, 1.19)	0.56 (0.42, 0.73)***	1.14 (0.87, 1.50)
2011-2012	0.91 (0.66, 1.25)	0.99 (0.70, 1.39)	1.05 (0.77, 1.44)
2013	N/A	0.70 (0.44, 1.13)	1.38 (0.84, 2.26)
2014	N/A	0.36 (0.19, 0.65)**	0.97 (0.61, 1.52)
2015	N/A	0.98 (0.48, 2.01)	1.35 (0.79, 2.31)

*N/A* Not available (question not asked during survey year)

* *p* < 0.05, ** *p* < 0.01, *** *p* < 0.001

## Discussion

These findings suggest that among California children 1 to 11 years of age, risk of asthma is higher among African Americans and lower for non-Hispanic Whites. Furthermore, asthma risk is higher among boys and older children. Diagnosis is also greater among those whose parents spoke English very well. The likelihood of diagnosis is lower among those with higher income, for children having no usual source of medical care, and for those living in a rural environment [32].

Across the seven asthma management and outcome measures in this study, parent English language proficiency was significantly associated with five of the measures. The consistent finding being that those with lower levels of proficiency were more likely to have the less desirable outcome. Usual source of care was also associated with five measures. Regarding socio-demographics, age was significantly associated with four measures, as was survey year. Parent education was significantly associated with three measures, race/ethnicity with two measures, and poverty level with one measure. Gender, rural/urban, insurance, and citizenship were not significantly associated with any measure.

For children diagnosed with asthma, those whose parents spoke English very well were more likely to have routine management measures, such as receipt of a written plan and daily medications and were less likely to have an emergency room visit in the past 12 months or to miss school due to asthma. Their parents were also much more likely to feel confident they could control asthma. Information plays a large role in controlling a chronic disease. Unfortunately prior analysis with CHIS data has shown that many California parents having children with asthma have suboptimal use of health literacy tools [17] and Latino adults with limited English proficiency report less confidence in filling out online forms [33]. Furthermore, a study outside of California found that parents with limited health literacy were more likely to have children whose asthma was not well controlled [34].

Others have found that asthma is a public health concern affecting minority groups and low-income children [35]. This study did not find an association between income and asthma management/outcome. This appears to be inconsistent with a study based on northern California Kaiser Permanente patients which found that lower-income parents who had higher cost-sharing were more likely to delay or avoid asthma care for their young children [36]. However, we were not able to evaluate delay in care or quality of medical care, other than giving an asthma management plan. The impact of poverty was most likely observed through usual source of care. Typically it is parents with steady jobs and/or higher income who can afford regular care at a doctor’s office. Being seen consistently in such a setting is associated with better care [37] and also is more likely to result in care through a medical home model [38] and to be following the latest treatment guidelines [39].

The higher rates among children in California compared to the United States as a whole [2] may reflect higher actual prevalence or suggest that perhaps better assessment and screening of asthma at the national level is necessary. The finding of higher asthma prevalence among boys and African Americans is also consistent with national data [2], as is the higher prevalence of those with two or more races [3]. California, due to its SCHIP (State Children’s Health Insurance Plan) which is primarily targeted towards immigrant minorities, could be one the reason for better diagnosis compared to national numbers [40]. Findings for Asians were also compable to national data by being similar to whites. Some study suggests higher rates of asthma of among Asian immigrants [41]; but any such effect in California is likely overcome by the dramatically lower likelihood of asthma among immigrants to the US. Others have found that minority children are more likely to have increased emergency visits, hospitalization and death from asthma [42], though we only found higher rates of emergency room visits for African Americans.

Thirty-six thousand students miss school every day because of asthma. Students having persistent asthma tend to show increased absenteeism and lower performances in standardized tests [42]. Therefore, school-based asthma education and management can be a potential asthma intervention [43]. Such programs should especially target and be tailored to families having limited English proficiency.

The prevalence of asthma seems to be lower among those of Mexican descent. However, this can be as a result of under diagnosis [44]. Limited English proficiency is another burden on some Hispanic families as they suffer poorer quality of care due to communication errors [45]. It is possible that low acculturation is not protective against asthma but that immigrants with little English proficiency are less likely to understand their physician or asthma symptoms, and thus report lower diagnosis rates [46]. In turn, this can overlook a critically underserved population, highlighting the need for culturally and linguistically tailored preventive measures. As such, there should be additional  public health efforts aimed at mitigating the current unmet asthma prevention and management care needs [47] for all families with limited English proficiency, particularlyLatinos. For example, delivery of health literacy interventions for caregivers of asthmatic children through culturally and linguistically competent clinicians could be of value [20].

### Limitations

The primary limitation of the study stems from its cross-sectional self-reported design. Such study designs do not provide insight into causation and therefore relative risks of outcomes cannot be evaluated. Moreover, self-reported data is prone to recall bias. Due to the cross-sectional nature of the survey, we were not able to formally test whether better management activities caused better outcomes. Our measure for ED visits was for all causes, not only asthma-related. Another limitation is selection bias, especially for families who do not have phones. Furthermore, results may not be generalizable outside of California. Despite such limitations, our findings have several strengths. The CHIS study was designed specifically to assess the health of Californians. In addition to the well-established sampling design, surveys are thorough in addressing child and family factors. Furthermore, the relatively large sample size allows for stable estimates.

## Conclusions

This large, population-based survey demonstrated that the English language proficiency of parents/caretakers had a stronger and more consistent effect on a group of asthma management measures than did health insurance, family income, and parental education. This highlights the importance of providing care and health education in a linguistically-appropriate manner for the most common chronic medical condition in children.

## Data Availability

The public-use data files are available from the University of California, Los Angeles, and were obtained through the following link: https://healthpolicy.ucla.edu/chis/data/Pages/public-use-data.aspx.
